# Deficits in pain medication in older adults with chronic pain receiving home care: A cross-sectional study in Germany

**DOI:** 10.1371/journal.pone.0229229

**Published:** 2020-02-21

**Authors:** Juliana Schneider, Engi Algharably, Andrea Budnick, Arlett Wenzel, Dagmar Dräger, Reinhold Kreutz

**Affiliations:** 1 Institute of Clinical Pharmacology and Toxicology, Charité - Universitätsmedizin Berlin, Corporate Member of Freie Universität Berlin, Humboldt-Universität zu Berlin, and Berlin Institute of Health, Berlin, Germany; 2 Institute of Medical Sociology and Rehabilitation Sciences, Charité - Universitätsmedizin Berlin, Corporate Member of Freie Universität Berlin, Humboldt-Universität zu Berlin, and Berlin Institute of Health, Berlin, Germany; Cleveland Clinic, UNITED STATES

## Abstract

**Objective:**

To analyze the pattern and appropriateness of pain medications in older adults receiving home care.

**Methods:**

We performed a prospective cross-sectional study in patients ≥65 years old having chronic pain and receiving home care in Berlin, Germany. Data on prescribed pain medications were collected using self-reported information, nursing documents, and medication plans during interviews at home. Pain intensity was determined with the numeric rating scale (NRS) and the Pain Assessment In Advanced dementia (PAINAD) scale. The Pain Medication Appropriateness Scale score (S_PMAS_) was applied to evaluate inappropriateness (i.e. a score ≤67) of pain medication.

**Results:**

Overall 322 patients with a mean age of 82.1 ± 7.4 years (71.4% females) were evaluated. The average pain intensity scores during the last 24 hours were 5.3 ± 2.1 and 2.3 ± 2.3 on NRS and PAINAD scale (range 0–10, respectively). Sixty (18.6%) patients did not receive any pain medication. Among the treated patients, dipyrone was the most frequently prescribed analgesic (71.4%), while 50.8% and 19.1% received systemic treatment with opioids and non-steroidal anti-inflammatory drugs, respectively. The observed median S_PMAS_ was 47.6 (range 0–100) with 58 (18.0%) of patients achieving appropriate values. Half of the patients were treated with scheduled, while 29.9% were only treated with on-demand medications. Cognitive status had no effect on appropriateness of pain treatment.

**Conclusions:**

We observed substantial deficits in dosing patterns and appropriateness of pain medication in older adults with pain receiving home care. This applied to both patients with and without severe cognitive impairment.

## Introduction

The global population has experienced a demographic change over the last century towards an aging population [1]. In Germany, an estimated 3.4 million individuals are in need of care and the majority of them (81%) are more than 65 years old, while 35% are at least 85 years old [2]. For this elderly population, pain represents a significant problem due to the high prevalence of musculoskeletal disorders, cancer, neuropathy and other medical conditions for which pain is a major symptom [3]. The prevalence estimates of chronic pain in the general population in Europe range from 12% to 30%, while in Germany a rate of 17% has been previously reported [4]. A more recent meta-analysis reported that about 62% of the population over the age of 75 years suffered from chronic pain in the UK indicating that the burden of chronic pain increases in line with aging [5]. On the other hand, a previous study indicated that there is an age-dependent discrepancy between the prevalence of chronic pain and pain interference or suffering from chronic pain [6]. Nevertheless, in the elderly it is estimated that about 70% of elderly individuals in home care are suffering from pain [7]. The problem is further complicated in those with cognitive impairment who are mostly incapable of communicating their own symptoms, which hinders appropriate management of pain in this population [8].

While pain itself is not a disease, rather a symptom to a multitude of underlying health disorders, chronic pain is regarded by some as a disease in its own right [9]. The implementation of a separate diagnostic code for chronic pain according to the newest International Classification of Diseases (ICD-11) underlines the need for better care for patients with chronic pain [10].

Uncontrolled pain substantially affects daily activities such as sleeping, housework and social relationships, and despite a plethora of available analgesic drugs, pain remains inadequately treated in most elderly patients [11, 12]. On the other hand, improved pain relief can positively reactivate a person’s physical and mental condition [13].

The elderly population is challenging in terms of its complexity and heterogeneity where comorbidity and polypharmacy complicate frailty [14]. Thus, improper use of pain medications and polypharmacy increases the risk of drug interactions and developing adverse drug reactions in the elderly [15]. The latter accounts for a great burden of disease in these patients including the need for hospital admission [16]. Hence, a recent meta-analysis showed that among patients admitted to hospital because of adverse drug reactions, non-steroidal anti-inflammatory drugs (NSAIDs) were frequently related to these admissions (percentages range from 2.3 to 33.3%) [16]. Optimal medical management and nursing care in pain treatment are thus essential to reduce morbidity and costs in long-term care. Nevertheless, to implement appropriate pharmacologic pain management in practice remains a challenging task.

We set out to assess the pattern of prescribed pain medications and their appropriateness in older adults receiving home care. We performed a prospective cross-sectional study in Berlin, Germany, and included patients independently from their cognitive status; thus patients with cognitive impairment were also enrolled.

## Methods

### Design and setting

The current analysis is a pre-specified analysis of the recently completed *ACHE* study (“Development of a Model for PAin Management in Older Adults ReCeiving Home CarE”) in Germany. *ACHE* is an observational cross-sectional study conducted in the home care setting in Berlin, Germany, from May 2017 to April 2019. The study complies with the declaration of Helsinki and was approved by the ethical committee of the Charité, Universitätsmedizin Berlin (EA1/368/14). Written informed consent was obtained by all the patients or their legal guardians in case of cognitive impairment.

### Study population

**Fig 1:**
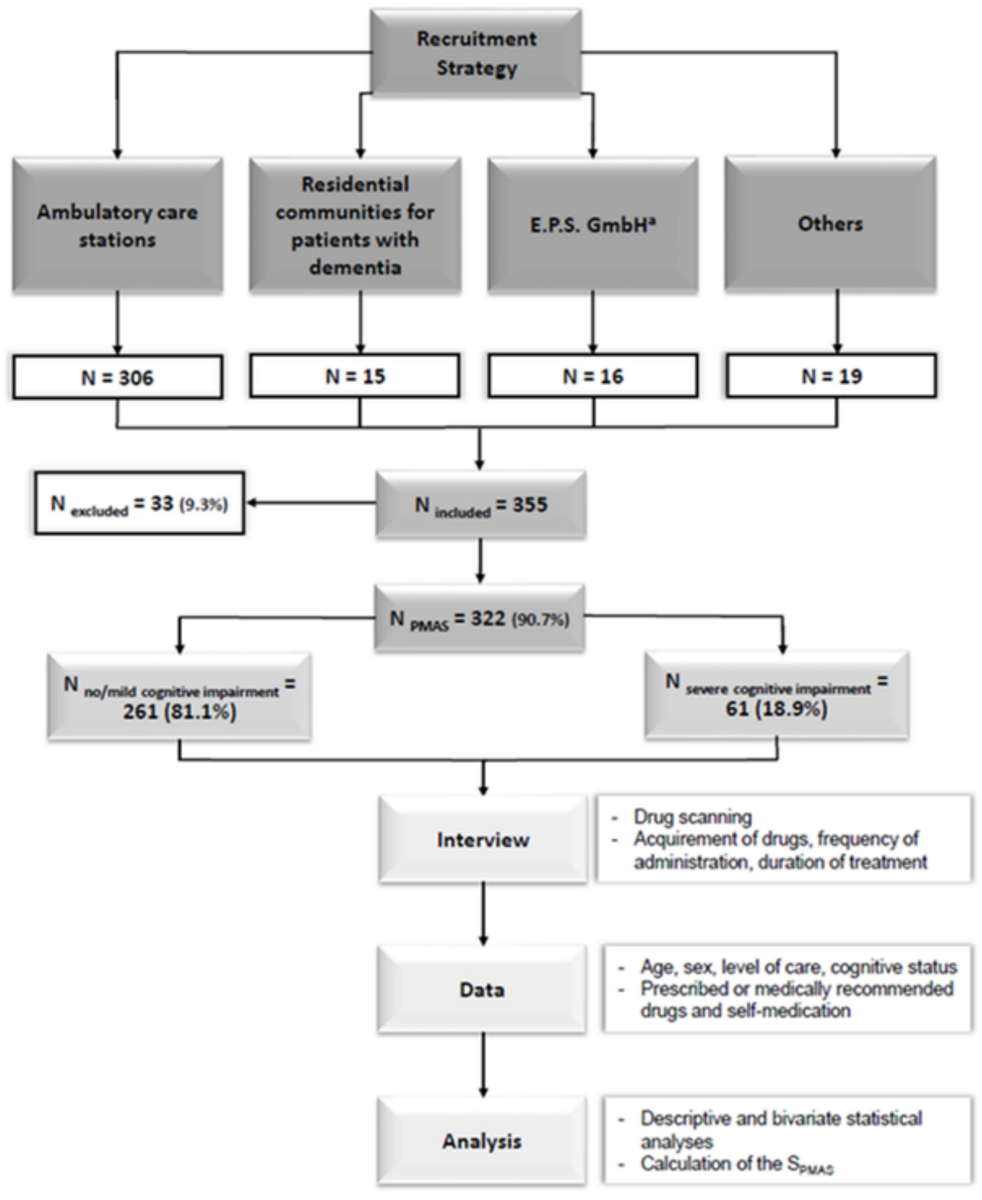
Flowchart—Recruitment strategy and methodical approach. S_PMAS_, Score on the Pain Medication Appropriateness Scale. ^a^E.P.S. GmbH is a service in Germany that provides advices to family caregivers regarding home care related issues.

Older adults receiving home care were mainly recruited through ambulatory nursing services (Fig 1) and were included if they met the following criteria: 1) aged 65 years or older; 2) suffering from chronic pain (≥ 3 months); 3) live at their own homes and 4) in need of care according to the legal regulations in Germany. Importantly, patients were enrolled independently from their cognitive status. Thus, we also included patients with cognitive impairment. There were 82 (15%) of 546 ambulatory care stations in Berlin that volunteered to take part in the study. The cognitive state of all patients was assessed using the Mini Mental Status Examination (MMSE) [17].

### Data collection

Data were obtained through face-to-face interviews in the patients’ own homes by five trained research assistants with different educational and professional qualifications including backgrounds in pharmacy, medical education, social science, nursing, or occupational therapy. Data on pain characteristics, pain management strategies, demographics as well as the level of care were collected and were based primarily on patients’ self-report, caregiving relatives, nurses and, if available, medication plans (Table 1). Drug-related data were systematically obtained by scanning medication packages using barcode scanners and the Instrument for Database-assisted Online recording for Medication (IDOM) [18]. The latter is based on detailed classification data provided by the AOK Research Institute (WIdO) that were updated on a monthly basis. All information regarding the active ingredients, the anatomical therapeutic chemical (ATC) classification, dosage, the mode of administration, “over the counter” (OTC)-drugs and nutritional supplements were recorded. Moreover, the investigators asked how patients obtained their drugs (e.g. by prescription, doctor’s recommendation or self-medication), the frequency of administration (scheduled or on-demand) as well as the duration of treatment. The gathered information about medications, diagnosis, and pain intensity, as well as pain relief by medications by the physician were utilized later to assess the appropriateness of pain management.

**Table 1:** Patient characteristics.

Characteristics	Full study population			PMAS population		
	Total N = 355	Women N = 254 (71.5%)	Men N = 101 (28.5%)	Total N = 322	Women N = 230 (71.4%)	Men N = 92 (28.6%)
**Age (years)**	82.2 ± 7.5	83.0 ± 7.1	80.4 ± 8.4	82.1 ± 7.4	82.7 ± 6.9	80.4 ± 8.3
**Care level (%)**^a^						
1	11.3	9.8	14.8	12.4	10.9	16.3
2	44.8	45.7	42.6	46.6	48.3	42.4
3	21.1	20.1	23.8	20.8	19.6	23.9
4	12.7	13.8	9.9	10.9	11.3	9.8
5	7.3	7.1	7.9	6.8	6.9	6.5
nd	2.8	3.5	1.0	2.5	3.0	1.1
**MMSE (%)**^b^^,^^c^						
0–17 points	22.6	23.7	19.8	18.9	19.5	17.4
18–23 points	15.8	15.4	16.8	15.8	17.0	13.0
24–30 points	61.6	60.9	63.4	65.3	63.5	69.6
**Barthel index**^d^^,^^e^	66.7 ± 27.5	66.9 ± 26.5	66.3 ± 29.9	68.7 ± 26.4	69.4 ± 24.9	67.0 ± 29.8

PMAS, Pain Medication Appropriateness Scale; nd, not determined; MMSE, Mini Mental State Examination.

^a^According to § 15 SGB XI, the level of care is based on the degree of self-dependence and ranges from 1 (lowest degree) to 5 (most severe impairment with special requirements for nursing care).

^b^The MMSE-score was calculated for 354 individuals.

^c^According to the MMSE classification [19]: 0–17 (severe cognitive impairment), 18–23 (mild cognitive impairment), 24–30 (no cognitive impairment).

^d^The Barthel-index was calculated for 349 individuals of the total population and for 319 of the PMAS population.

^e^The motor function restriction is graded by the Barthel index into [20]: 0–15 (very severe), 20–30 (severe), 40–55 (intermediate severe), 60–75 (intermediate), 80–95 (low) and 100 (no or minimal functional impairment).

### Instruments and measures

Pain management was evaluated by using the Pain Medication Appropriateness Scale (i.e. PMAS) originally designed to detect problems in pain therapy in nursing homes [21]. The PMAS is a valid tool to analyze the pharmacological treatment of pain. To check for scale reliability, Cronbach´s coefficient alpha was calculated for PMAS [22]. This scale consists of ten items allocated to five main domains (appropriate medication for pain syndrome, scheduled dose interval, titration of medication to severity of pain including the pain management index (PMI), constipation prevention, and exclusion of geriatric high-risk drugs). In a previous study, the PMAS was successfully adapted for the evaluation of pain medication management in Germany [23]. For the evaluation of PMAS, pain intensity was assessed using numeric rating scales (NRS) for pain as implemented within the Brief Pain Inventory (BPI) [24] in patients without cognitive impairment. In patients with an MMSE value <10 or in whom NRS could not be evaluated for other reasons, the Pain Assessment In Advanced Dementia (PAINAD) scale [25] was applied. The corresponding validated German transcript for the PAINAD scale was used [26]. The BPI includes four NRS ranging from 0 (no pain) to 10 (worst imaginable pain) to asses four items of pain intensity (worst pain, lowest pain, average pain, current pain) over the past 24 hours.

The German PAINAD scale consists of five items that focus on characteristic behavior due to pain in patients with advanced dementia as a physical indication of pain suffering (breathing, negative vocalization, facial expression, body language and consolability). For each item considering different behavioral patterns, there is a scale from 0 to 2 and total scores between 0 and 10 are possible.

A checklist for special types of pain was also applied. Functional status was evaluated by the Barthel-Index (BI) [27, 28].

Only medications that were prescribed by the treating physicians were considered for PMAS analysis, including both scheduled and on-demand medications; dosing intervals were also considered. Furthermore, we adapted the PMAS according to current national guidelines of pain management as well as high risk drugs avoided in geriatric patients [29, 30] (S1 Table). In addition, we formulated a four-class categorization of the PAINAD-score using boxplots and substantiated our approach by the Receiving Operating Characteristic (ROC)-curve analysis (S2 Table and S1 Fig) [31].

Each item of the PMAS was assessed if it applied to the patient’s individual situation. As a result, there are different maximum points possible. The final PMAS-score (i.e. S_PMAS_) reflects a percentage considering the possible points (S_possible_), as well as the applicable points (S_total_) according to the formula [21]:

$$S_{PMAS}=\sum (S_{total})/\sum (S_{possible})*100$$

An S_PMAS_ ≤67 value indicates inappropriate pain medication as suggested [21]. In individuals in whom self-reported pain assessment was not feasible, a score of ≥1 on the PAINAD scale indicated probable pain.

### Data analysis

Descriptive statistics were used to describe demographics of patients and variables related to pain- and medications. Data were analyzed using IBM SPSS Statistics, version 25 (IBM Corp, Armonk, NY). The analysis of S_PMAS_ is based on an adapted version of the reported German version of the PMAS [23]. The distribution of variables was checked using Shapiro-Wilks test. For data without normal distribution, the non-parametric Mann-Whitney U test or Kruskal-Wallis H test were used as appropriate. Thus, the latter was used to compare S_PMAS_ values of the different subgroups related to the mode of drug intake (only on demand, only scheduled, both, none) and was followed by Dunn-Bonferroni test for posthoc analysis. Data for S_PMAS_ were presented as median and range. Spearman’s correlation and Chi-squared test were conducted to check associations between patients`characteristics and the S_PMAS_. Statistical significance was determined with an alpha value of 0.05.

## Results

### Study participants

A total of 355 patients (mean age 82.2 ± 7.5 years, 71.5% females) met the formal inclusion criteria of the overall *ACHE* study; data of 322 patients (mean age 82.1 ± 7.4 years, 71.4% females) were available for analysis of appropriateness of pain medication, i.e. PMAS population (Table 1). Patients were excluded because of missing data regarding medication, diagnosis or some other aspects that are necessary to calculate S_PMAS_. The majority of patients (46.6%) received the second level of care, while for 2.5% the level of care was not determined (Table 1). No or only mild cognitive impairment was observed in 261 (81.1%) patients, while 18.9% had severe cognitive impairment (MMSE ≤17 points). The mean Barthel index was 68.7 ± 26.4. All patients suffered from chronic pain and had an average pain intensity score of 5.3 ± 2.1 on the NRS (range 0–10) during the last 24 hours. The corresponding score was 2.3 ± 2.3 on the PAINAD scale (range 0–10) in patients with cognitive impairment (n = 64). Overall 211 (65.5%) patients reported current pain at the time of interviewing with an average intensity of 5.7 ± 1.9 on the NRS and 3.0 ± 2.2 on the PAINAD scale in the last 24 hours. The mean score for worst pain that was obtained in patients with current pain and without cognitive impairment over the past 24 hours was 6.9 ± 2.1. Almost half of the patients (n = 155) have had chronic pain for at least 10 years. Low back pain (75.8%), osteoarthritis (67.2%) and neuropathic pain (57.1%) were the most frequently recorded underlying pain conditions, besides other diseases such as headache (32.9%), rheumatoid arthritis (14.2%) and urarthritis (12.9%).

### Pattern of pain medications

Overall sixty (18.6%) patients did not receive any pain medication and from the 211 patients who reported having current pain during the interview, 37 (17.5%) received no prescribed pain medication. About half of the patients (162/322) were treated with systemically administered scheduled analgesics. About a quarter of patients (81/322) were only treated with scheduled and 29.9% (96/322) received only on-demand medications.

**Fig 2:**
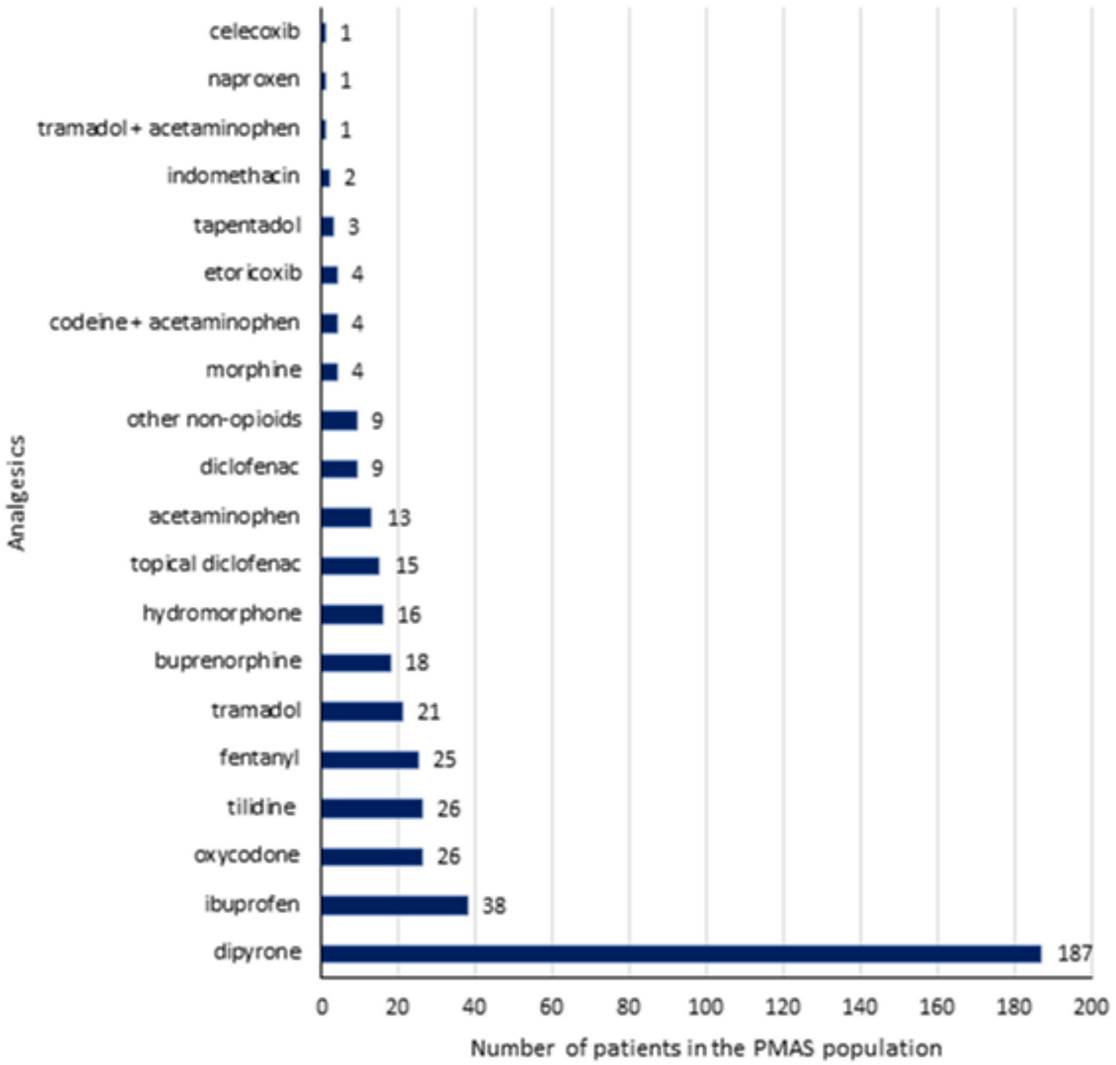
Numbers of individuals with prescribed analgesics among the PMAS population. PMAS, Pain Medication Appropriateness Scale.

Dipyrone was most frequently prescribed (Fig 2) in a total of 187 (71.4%) of treated patients of whom 81 (43.3%) patients received dipyrone as monotherapy. The second most frequently prescribed drug was ibuprofen in 38 (14.5%) of treated patients. Only 50 (19.1%) of all treated patients received systemic treatment with any NSAID, either as scheduled or as on-demand medication. Overall 133 (50.8%) patients received treatment with systemic opioids most frequently as scheduled treatment (n = 118), but only 45.8% of the latter were prescribed additional treatment with laxatives for constipation prophylaxis.

### Appropriateness of pain medications

The observed median S_PMAS_ was 47.6 (range 0–100). The PMAS was reliable with a value of 0.83 for Cronbach´s alpha. According to the suggested cutoff of ≤67 [21], only 58/322 (18.0%) of patients received adequate pain medication. S_PMAS_ in patients with prescribed analgesics was significantly higher (median: 53.3 [range 0–100]) than in subjects without any pain medication (median: 6.7 [range 0–66.7], Mann Whitney test, *U = 1100*.*5*, p < 0.001). Patients who received only on-demand pain medication achieved lower S_PMAS_ values compared to patients treated only with scheduled analgesics (median: 33.3 [range 0–100] vs. 50.0 [range 22.2–83.3], Kruskal-Wallis H test, H = 197.3, p < 0.001). Patients managed by both scheduled and on-demand medication (n = 84) obtained the highest S_PMAS_ (median: 71.4 [44.4–93.3], Kruskal-Wallis H test, p < 0.001). Age, sex, cognitive state, school education, professional qualification, functional state, and pain intensity did not significantly affect appropriateness of pain medication.

**Fig 3:**
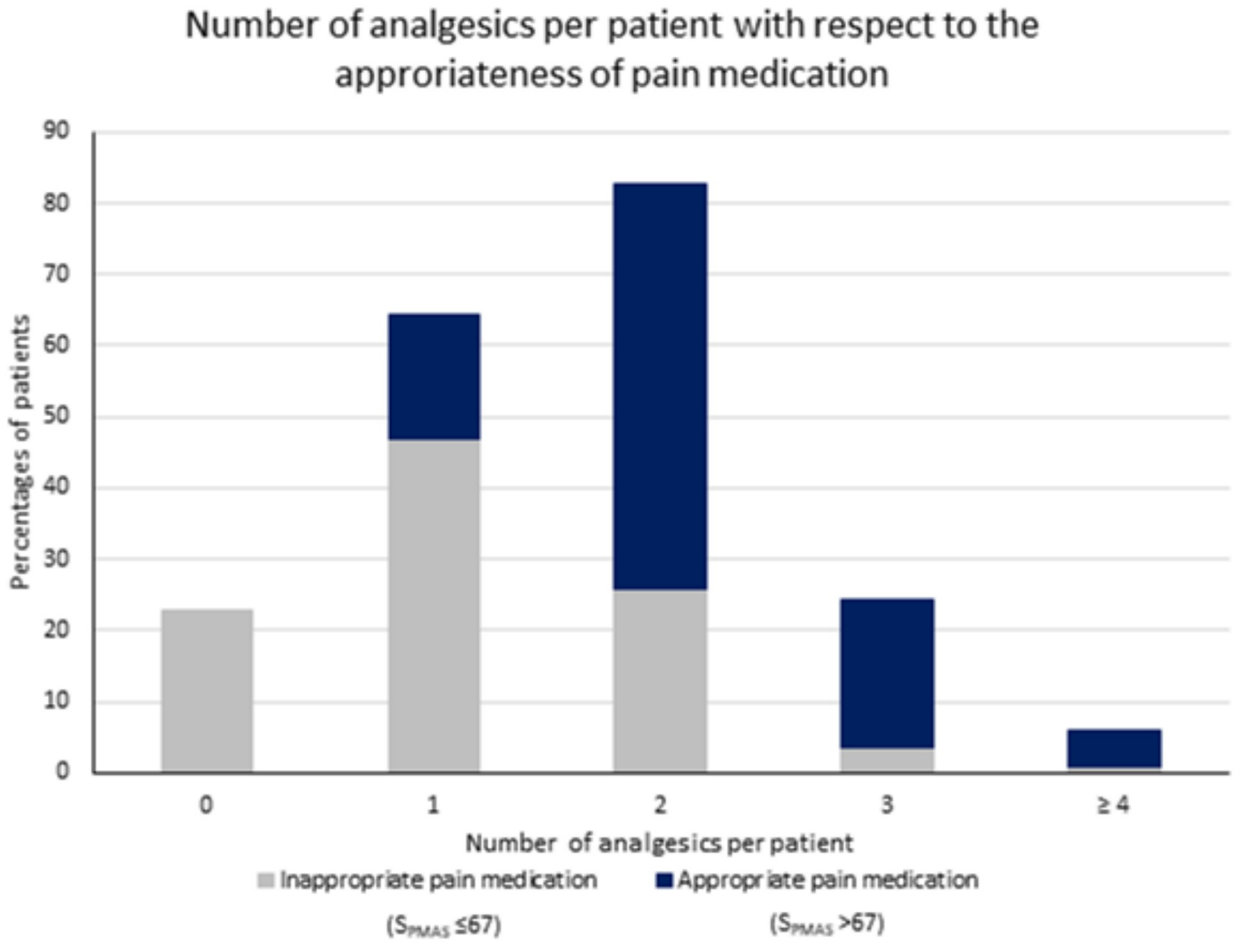
Number of analgesics per patient with respect to the appropriateness of pain medication. S_PMAS_, Score on the Pain Medication Appropriateness Scale.

We observed a moderate correlation between the number of prescribed analgesics and S_PMAS_ (r = 0.672; p < 0.001). Patients who achieved an S_PMAS_ ≤67 were treated with an average of 1.1 ± 0.8 (range 0–5) analgesic drugs, while patients with an S_PMAS_ >67 received 2.1 ± 0.8 (range 1–4) medications. A total of 134 (51.1%) patients received only one analgesic, the majority of them had an S_PMAS_ ≤67. Nevertheless, there were 10/58 (17.2%) patients who were adequately treated with monotherapy (Fig 3).

## Discussion

In the current cross-sectional study, we identified several important deficits in pain medication treatment in older patients receiving home care in Germany. First of all, 18.6% of patients with a history of chronic pain did not receive any pain medication. Secondly, a substantial number of patients were, in contrast with guideline recommendations [32], only treated with either scheduled (25.2%) or on-demand medications (29.9%). This is important against the background of the history of chronic pain and intensity of current pain as observed during the last 24 hours in our cohort of patients. According to the *MOBILIZE Boston* study [33], about 30% of community-living older adults with moderate to severe pain were also inadequately treated, while 50% did not receive any pain medication [33]. The latter finding might be explained by the relative high ratio of patients with very mild to mild pain enrolled in this study [33]. Roy et al. reported in agreement with our current findings, that 16% of institutionalized elderly patients with pain did not receive treatment with analgesics [34]. We also found in a previous study in the nursing home setting in Germany, that 20.6% of residents with chronic pain received no treatment with pain medications [35].

In agreement with previous findings in Germany [23, 35], dipyrone was by far the most frequently prescribed analgesic in the current study. Despite the well-known risk of agranulocytosis associated with dipyrone [36], the use of this drug seems well justified particularly in the vulnerable elderly population, because of its favorable overall risk-benefit profile as compared to NSAIDs [37, 38]. Although ibuprofen was the second most frequently prescribed drug in our study, the overall prescription rate of systemic NSAIDs was relatively low (19.1%). Their use is rated negatively in the evaluation of the appropriateness of pain medication in the PMAS tool when prescribed as scheduled medication for a period longer than four weeks. Furthermore, according to the Fit fOr The Aged (FORTA) List, NSAIDs should be generally avoided in the elderly [30]. However, the use of acetaminophen, that is often preferred in elderly patients due to its better safety profile [39], was also very low (5.0%). The latter could be related to the fact that acetaminophen prescriptions in Germany are only reimbursed by health insurances in patients suffering from severe pain and who are treated with opioids [40]. On the other hand, a substantial fraction (50.8%) of treated patients in our study was treated with opioids. This could be ascribed to the observed high prevalence of patients with osteoarthritis, low back pain and neuropathic pain in whom opioids are often recommended [32]. However, the use of opioids for persistent pain is not without limitations particularly in the treatment of older adults because of changes in their pharmacodynamic and pharmacokinetic profile that may require dose adjustments [41]. Their long-term use may result in serious adverse effects such as sedation, impaired balance and falls [41] in the vulnerable elderly population exposed to polypharmacy [42].

When considering appropriateness of pain medication, less than one fifth (18%) of patients received adequate pain treatment according to the suggested S_PMAS_ cutoff value >67 [21]. Our results are thus consistent with a corresponding study in the nursing home setting in Germany that reported also deficits in pain treatment, although with a somewhat higher percentage of patients (i.e. 24%) receiving appropriate treatment [23, 43]. A more recent study by Rabenberg et al. substantiated our results by reporting deficits in pain treatment in the elderly [44]. In their study, one out of ten older patients had a problem (under- or over-treatment) with pain medications [44].

One strength of our study is related to the fact that we included also patients with severe cognitive impairment (18.9%). This is in contrast to previous studies that either included only a very small number of these patients [21] or excluded patients with moderate to severe cognitive impairment [33] or dementia [45]. In order to assess the appropriateness of pain medication in the patients with severe cognitive impairment, we used a four-class categorization of the PAINAD-score to assess pain severity in this group of patients with a cutoff value of 1 for mild pain (S3 Table). In the literature, a cutoff score of 2 on the PAINAD scale indicates likely pain in patients with dementia, nevertheless, pain cannot be ruled out with a score less than 2 for cognitively impaired individuals [46].

The observed positive correlation between the number of analgesics and S_PMAS_ in the current study is not surprising. A combination of two or more analgesics with complementary mechanisms of action is projected to provide greater pain relief [32]. However, this does not always imply that patients with the highest number of analgesics are treated best. It is equally important to consider the class of drug in relation to the pain condition, the dosage, dosing interval and the mode of application that also affect appropriateness within the evaluation using PMAS. Indeed, there were patients in our sample treated with analgesic monotherapy who reached the threshold for appropriate treatment (S_PMAS_ >67). In addition, the combined prescription of fast-onset, short-acting, on-demand analgesics with scheduled analgesics for breakthrough pain is useful for optimal pain control [32]. This is corroborated by our finding where patients treated with both scheduled and on-demand analgesics reached the highest S_PMAS_. In analogy to regularly-administered medications, clear information regarding the dose (initial and maintenance), the dosing interval and the duration of treatment should be provided to patients when on-demand medications are prescribed [47].

As a case in point, we noticed that for 40.6% of prescribed on-demand analgesics, the dosing interval was unknown. In these cases, we could not assess whether these analgesics were adequately dosed by physicians. As a result, no additional points for adequate dosing intervals were considered during evaluation.

Our study has some limitations. First, we analyzed only a relatively small sample because access to this study population is very difficult to achieve in Germany. Second, the patients were interviewed/observed only once, i.e. at a single occasion. Third, no interrater reliability validity was done in our study. However, interrater reliabilities in our previous studies using a similar overall approach were found to be satisfactory and highly significant [23, 35]. The PMAS has also some limitations as previously pointed out [21, 23, 35]. Thus, in the calculation of the S_PMAS_, non-pharmacological pain treatment is not considered. The potential of this treatment modality should not be underestimated, especially in the elderly where side effects of medications, drug-drug interactions, and comorbidities can impede the use of pharmacological treatments [48]. The combination of non-pharmacological and pharmacological pain management is important for effective pain relief [14, 49]. Furthermore, no points are considered for treatment with co-analgesic drugs. Although co-analgesics are in general not primarily indicated to treat pain, they are efficacious when combined with other analgesics [32, 50] and may also be prescribed as monotherapy for special pain syndromes [32]. Nevertheless, the PMAS tool is best known for its reliability and flexibility [43], whereby items could be eliminated during assessment if they do not apply to individual patients. Accordingly, we modified this scale in agreement with current recommendations regarding the use of cannabinoids in the treatment of chronic pain [51, 52]. In addition, a moderate to high level for scale reliability for the PMAS was indicated by a Cronbach´s alpha value of 0.83 [22].

## Conclusions and implications

We observed substantial deficits related to lack of treatment, inadequate dosing patterns and overall high frequency of inappropriate use of pain medications in older adults with pain receiving home care. Therefore, interventional strategies to improve treatment by implementing a multidisciplinary network approach involving physicians, pharmacists, nurses and patients, possibly supported by modern eHealth tools [53] is highly warranted.

## Supporting information

S1 Fig Box plot used for the four-class categorization of the PAINAD-score. In consideration of all patients for whom the PAINAD sum score was available (n = 81), an appropriate four class categorization was not possible (A). For further explorative data analyses, we excluded patients with a total sum score of 0 on the PAINAD-scale (n = 57). Thus, we got a boxplot indicating four classes of the PAINAD sum score (B) as described in S2 Table.
(DOCX)

S1 Table Modification of the German version of the pain medication appropriateness scale (PMAS). PMAS, Pain Medication Appropriateness Scale; NSAIDs, non-steroidal anti-inflammatory drugs; PRISCUS list, Potentially Inappropriate Medications in the Elderly.
(DOCX)

S2 Table Four class categorizations of the PAINAD-score. (DOCX)

S3 Table The PAINAD-score cutoff according to ROC curve. With regard to the highest level of sensitivity and specificity, a PAINAD score greater than 0.5 was chosen to determine cognitively impaired patients with pain-associated physical expressions. ROC, Receiving Operating Characteristic.
(DOCX)
