# Supplementary material for: Deficits in pain medication in older adults with chronic pain receiving home care: A cross-sectional study in Germany
Source: PLoS One. 2020 Feb 21;15(2):e0229229. doi: 10.1371/journal.pone.0229229 (PMC7034806; doi:10.1371/journal.pone.0229229)
Supplement: S1 Fig — In consideration of all patients for whom the PAINAD sum score was available (n = 81), an appropriate four class categorization was not possible (A). For further explorative data analyses, we excluded patients with a total sum score of 0 on the PAINAD-scale (n = 57). Thus, we got a boxplot indicating four classes of the PAINAD sum score (B) as described in S2 Table. (DOCX) [file pone.0229229.s001.docx]

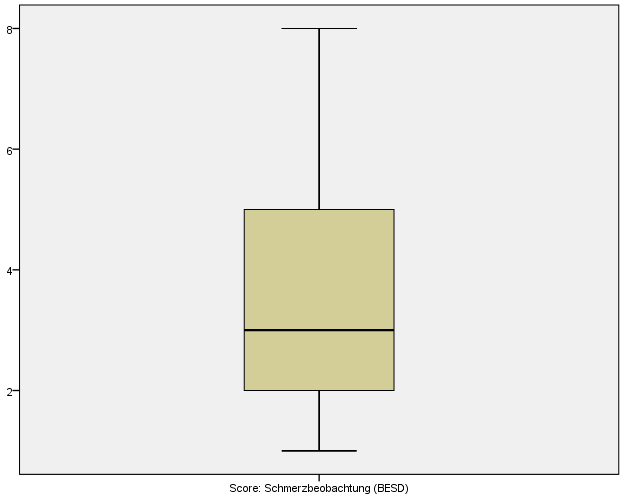


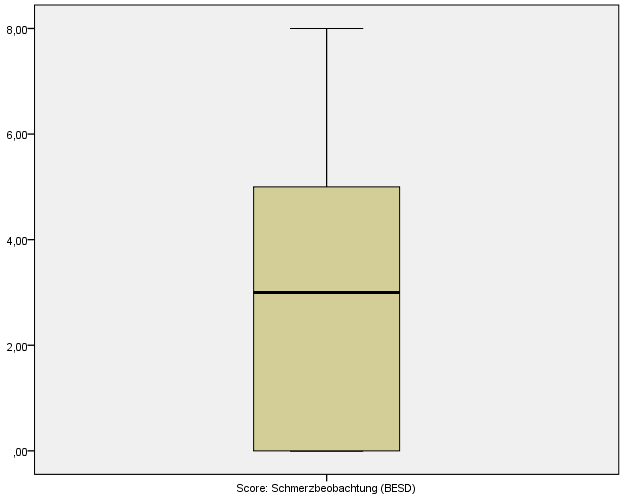


**A**

**B**

**S1 Figure.** Box plot used for the four-class categorization of the PAINAD-score.

In consideration of all patients for whom the PAINAD sum score was available (n=81), an appropriate four class categorization was not possible (A). For further explorative data analyses, we excluded patients with a total sum score of 0 on the PAINAD-scale (n=57). Thus, we got a boxplot indicating four classes of the PAINAD sum score (B) as described in S2 Table.
