# Supplementary material for: Deficits in pain medication in older adults with chronic pain receiving home care: A cross-sectional study in Germany
Source: PLoS One. 2020 Feb 21;15(2):e0229229. doi: 10.1371/journal.pone.0229229 (PMC7034806; doi:10.1371/journal.pone.0229229)
Supplement: S1 Table — PMAS, Pain Medication Appropriateness Scale; NSAIDs, non-steroidal anti-inflammatory drugs; PRISCUS list, Potentially Inappropriate Medications in the Elderly. (DOCX) [file pone.0229229.s002.docx]

**S1 Table.** Modification of the German version of the Pain Medication Appropriateness Scale (PMAS).

| **Item on the PMAS** | **Modification** |
| --- | --- |
| Item 2. Neuropathic pain: | - Points were also possible for capsaicin and cannabinoids. |
| Item 6. Pain severity – calculation of the Pain Management Index (PMI): | - Cannabinoids were considered as non-opioid analgesics and were scored with 1 point. - Tapentadol was considered as a strong opioid and was scored with 3 points. |
| Item 9. Appropriate constipation prevention with scheduled opioids: | - It is possible, to add points to reach 3/3 points. |
| Item 10. Drugs to avoid in geriatric patients: | - - 1 point for routine dosing of NSAIDs excluding topical NSAIDs - - 1 point for antiarrhythmic drugs of class 1 and sotalol - - 1 point for clonidine excluding topical clonidine - - 1 point for H_1_-antihistamines according to PRISCUS list - - 1 point for promethazine and strong sedative neuroleptics excluding melperone and pipamperon - - 1 point for anticholinergic muscle relaxants or antispasmodics (orphenadrine, hyoscyamine, atropine, oxybutynin, solifenacin, tolterodine (not retarded), baclofen and tetrazepam) |

PMAS, Pain Medication Appropriateness Scale; NSAIDs, non-steroidal anti-inflammatory drugs; PRISCUS list, Potentially Inappropriate Medications in the Elderly.
