# Supplementary material for: Deficits in pain medication in older adults with chronic pain receiving home care: A cross-sectional study in Germany
Source: PLoS One. 2020 Feb 21;15(2):e0229229. doi: 10.1371/journal.pone.0229229 (PMC7034806; doi:10.1371/journal.pone.0229229)
Supplement: S2 Table — (DOCX) [file pone.0229229.s003.docx]

**S2 Table.** Four class categorizations of the PAINAD-score.

| **Categories** | **Pain severity** |
| --- | --- |
| 0 | No observed pain pattern |
| 1 - 2 | Mild pain |
| 3 - 4 | Moderate pain |
| ≥ 5 | Severe pain |
