# Supplementary material for: Deficits in pain medication in older adults with chronic pain receiving home care: A cross-sectional study in Germany
Source: PLoS One. 2020 Feb 21;15(2):e0229229. doi: 10.1371/journal.pone.0229229 (PMC7034806; doi:10.1371/journal.pone.0229229)
Supplement: S3 Table — With regard to the highest level of sensitivity and specificity, a PAINAD score greater than 0.5 was chosen to determine cognitively impaired patients with pain-associated physical expressions. ROC, Receiving Operating Characteristic. (DOCX) [file pone.0229229.s004.docx]

**S3 Table.** The PAINAD-score cutoff according to ROC curve.

| **Positive, if greater than or equal to a.** | **Sensitivity** | **1 - Specificity** |
| --- | --- | --- |
| -1,00 | 1,000 | 1,000 |
| ,50 | 1,000 | ,400 |
| 1,50 | 1,000 | ,225 |
| 2,50 | 1,000 | ,000 |
| 3,50 | ,683 | ,000 |
| 4,50 | ,561 | ,000 |
| 5,50 | ,341 | ,000 |
| 6,50 | ,146 | ,000 |
| 7,50 | ,098 | ,000 |
| 9,00 | ,000 | ,000 |

a. The smallest cutoff value is the minimum observed rest value minus 1, and the largest cutoff value is the maximum observed test value plus 1. All the other cutoff values are the averages of two consecutive ordered observed test values.

With regard to the highest level of sensitivity and specificity, a PAINAD score greater than 0.5 was chosen to determine cognitively impaired patients with pain-associated physical expressions. ROC, Receiving Operating Characteristic.
